# Tuning filament composition and microstructure of 3D-printed bioceramic scaffolds facilitate bone defect regeneration and repair

**DOI:** 10.1093/rb/rbab007

**Published:** 2021-03-13

**Authors:** Yi Chen, Jiaping Huang, Jiamei Liu, Yingming Wei, Xianyan Yang, Lihong Lei, Lili Chen, Yanmin Wu, Zhongru Gou

**Affiliations:** 1 Department of Stomotology, The Second Affiliated Hospital of Zhejiang University School of Medicine, Hangzhou 310009, China; 2 Zhejiang-California International Nanosystems Institute, Zhejiang University, Hangzhou 310058, China

**Keywords:** core–shell-typed pore filament, component distribution, microporous structures, controllable degradation, 3D printing

## Abstract

It is still a challenge to optimize the component distribution and microporous structures in scaffolds for tailoring biodegradation (ion releasing) and enhancing bone defect repair within an expected time stage. Herein, the core–shell-typed nonstoichiometric wollastonite (4% and 10% Mg-doping calcium silicate; CSiMg4, CSiMg10) macroporous scaffolds with microporous shells (adding ∼10 μm PS microspheres into shell-layer slurry) were fabricated via 3D printing. The initial mechanical properties and bio-dissolution (ion releasing) *in vitro*, and osteogenic capacity *in vivo* of the bioceramic scaffolds were evaluated systematically. It was shown that endowing high-density micropores in the sparingly dissolvable CSiMg10 or dissolvable CSiMg4 shell layer inevitably led to nearly 30% reduction of compressive strength, but such micropores could readily tune the ion release behaviour of the scaffolds (CSiMg4@CSiMg10 vs. CSiMg4@CSiMg10-p; CSiMg10@CSiMg4 vs. CSiMg10@CSiMg4-p). Based on the in rabbit femoral bone defect repair model, the 3D μCT reconstruction and histological observation demonstrated that the CSiMg4@CSiMg10-p scaffolds displayed markedly higher osteogenic capability than the other scaffolds after 12 weeks of implantation. It demonstrated that core–shell bioceramic 3D printing technique can be developed to fabricate single-phase or biphasic bioactive ceramic scaffolds with accurately tailored filament biodegradation for promoting bone defect regeneration and repair in some specific pathological conditions.

## Introduction 

In the recent years, unprecedented growth in bone damages and diseases enables scientists to fabricate bioresorbable, osteoconductive and/or osteostimulative bone implants [1, 2]. To achieve ideal results in bone regeneration and repair, sufficient quantity and quality of new bone augmentation are the predominant factors in the so-called porous scaffold materials for enough biomechanical support with a balanced time schedule [3]. Great effort has been made over the past several decades in searching for biomaterials for bone repair applications. In particular, the inorganic Ca-phosphate and Ca-silicate biomaterials are the materials of choice used in the most surgical implant operations in the past decades [4–6]. However, it is still a great challenge to enhance bone repair progress and material biodegradation in critically sized defects within a narrow ‘time window’, especially the use of sparingly dissolvable hydroxyapatite bioceramics and other conventional biphasic Ca-phosphate biomaterials [7, 8].

Among the Ca-silicate bioceramics, wollastonite (CaSi) is considered as a promising candidate due to its high bioactivity and good biocompatibility [9–11]. The calcium and silicon released from CaSi scaffolds can promote cell proliferation and differentiation [12]. Meanwhile, CSi-containing biomaterials soaked in simulated body fluids (SBF) can readily form biomimetic apatite on the surface and promote bone regeneration in animal bone defect models [9, 13]. Unfortunately, the low mechanical stability and ultra-fast bio-dissolution rate of pure CSi bioceramic scaffolds have been demonstrated to be unfavourable for new bone tissue ingrowth *in vivo* [14]. Our previous studies have shown that, however, a series of dilute magnesium doping CSi (CSiMg*x*, *x *=* *3 − 14%) bioceramics could improve mechanical properties, tune biodegradation rate and promote the surface biocompatibility and osteogenesis [15, 16]. Magnesium is the fourth abundant element in human body, which affects the activity of osteoblasts and the mineralization of bone tissue to improve bone growth [17].

It is agreed that the use of biphasic mixing, or dopants to control the behaviour of materials stands on the heart of biomaterials, which would otherwise be far from its performance requirements [12, 18, 19]. The traditional biphasic hybrid approach is thought to readily compromise the biodegradable and bioactive properties of single-phase bioceramic, yet the spatiotemporal evolution of pore networks and biodegradation of composite bioceramics cannot be flexibly tailored and controlled [3, 7, 20]. In this aspect, we have recently developed a one-step scalable manufacturing route to prepare the core–shell-typed biphasic bioceramic microspheres with gradient distribution using coaxially arranged capillary systems, which readily controls the microstructure in the specific core or shell component to fully utilize advantages of component individual [21, 22]. In addition, the core–shell filament design has time-dependent physicochemical properties [23], whilst the effectiveness of the core–shell-structured filament in bioceramic scaffold and its biological performances *in vivo* need to be further explored.

On the other hand, the factors influencing bone regeneration and repair include not only the chemical composition but also the pore characteristics of the porous scaffolds [24]. A series of previous studies have shown that not only macropores of the porous scaffold play an important role in bone engineering, but the micropores are beneficial for bone regeneration through increasing protein adsorption and release of degradation products and also provide additional space for bone ingrowth with time [25, 26]. 3D printing is a cutting-edge technology to develop porous scaffolds with complex and well-defined geometric shapes that allow for the flexible choice of custom geometric sizes and materials to create 3D porous structures [27]. Recent studies have reported 3D-printed bioceramic scaffolds with microporous strands are beneficial for bone regeneration [28, 29]. However, there is no research related to the dynamic adjustment of the spatiotemporal evolution of the microporous structures of bioceramics. Thus, the relationship between bioactive ion release/micropores evolution of bioceramic scaffold and stimulating osteogenic efficacy is highly valuable for exploring in critical size bone defect conditions.

Under the above considerations, we developed the core–shell-typed nonstoichiometric CSi bioceramic scaffolds based on the ceramic ink writing technique with coaxial double nozzle system, using 4% and 10% Mg-doped CSi bioceramic (CSiMg4, CSiMg10) powder as raw materials. To further understand the effect of microporous structure on the physicochemical characteristics and osteostimulative capability of scaffolds, polystyrene (PS) microsphere porogens (∼10 μm in diameter) were added into the shell-layer slurry before direct in writing. The microstructure, mechanical properties, bio-dissolution *in vitro* and osteogenesis *in vivo* of these core–shell scaffolds were evaluated, respectively.

## Materials and methods

### Preparation of CSiMg4 and CSiMg10 powders

The nCSi powders including CSiMg4 and CSiMg10 were prepared following the wet chemical method described previously [15]. Briefly, the mixture (2.0 l) of Ca(NO_3_)_2_ and Mg(NO_3_)_2_ (0.5 M; pH 9–10) with a Ca^2+^/Mg^2+^ molar ratio of 96:4 or 90:10 was prepared and then added dropwise into 2.0 lNa_2_SiO_3_ solution (0.5 M) under stirring. The precipitate powders were dried at 85°C for 24 h. After calcined at 850°C for 3 h, the bioceramic powders were ground by zirconia ball media in ethanol for 5 h.

### 3D printing core–shell bioceramic scaffolds

**Scheme 1.**
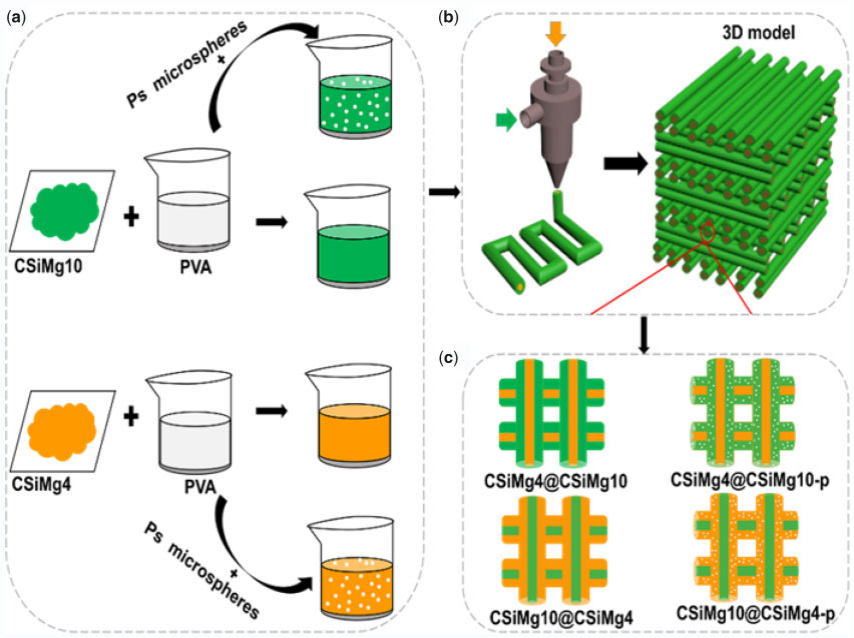
Schematic diagram of preparation of three-dimensional porous core-shell scaffolds. Preparation of bioceramic slurries (a); 3D printing and 3D scaffolds with core-shell strut structure (b); the core-shell-typed nCSi bioceramic struts in the four groups of scaffolds (c).

The CSiMg4 and CSiMg10 powders were dispersed in 6.0 wt% polyvinyl alcohol (PVA, Sigma-Aldrich, St. Louis, MO) aqueous solution （6.4 g/8.0 ml） separately under gentle stirring. The polystyrene (PS) granules of ∼10 μm in diameter were mixed with the CaSiMg4/PVA or CSiMg10/PVA slurry with a PS-to-powder mass fraction of 20% (Scheme 1a). Different slurries were simultaneously extruded into coaxial line deposition through core or shell nozzles, and the deposition angle after forming two layers was changed from 0° to 90°. The spacing between filaments and layer height were set as 500 and 1000 μm, respectively. The slurry of core layer and shell layer was injected into 1-ml syringe, respectively, and then connected with a 3D printer (Printnovo-3, Plino Technology, Hangzhou, China) and coaxial double-nozzle system. The extrusion speed of core layer and shell layer was 1.5 and 8 mm/s, respectively. The temperature of printing platform was maintained at nearly 33°C. Then, the core–shell-filament bioceramic scaffolds (6.5 × 5 × 5 mm) were manufactured with the 3D printer and a coaxial bi-nozzle needle with an inner diameter of 0.5 mm and an outer diameter of 0.9 mm by depositing strands in a layer-by-layer fashion (Scheme 1b), a constant printing speed of 15 mm/s. Subsequently, the wrote porous scaffolds were dried at 80°C for 24 h and then sintered at 1100°C using a heating rate of 3°C/min and held at the target temperature for 3 h before cooling. The sintered scaffolds with and without porous shell (Scheme 1c) were denoted as CSiMg4@CSiMg10, CSiMg4@CSiMg10-p, CSiMg10@CSiMg4 and CSiMg10@CSiMg4-p.

### Phase and microstructure analysis

The elemental composition (Ca, Si, Mg) of the bioceramic powders was measured by inductively coupled plasma-optical emission spectrometry (ICP-OES; Thermo, Waltham, MA). The phase composition of the powders was analysed using X-ray diffraction (XRD; Rigaku, Tokyo, Japan) with a Cu*K*α radiation (2°/min). The particle size measurements were carried out using the dynamic light scattering technique and particle size distribution analysis assuming spherical shape (LDSA; ZETASIZER, Nano S90, Worcestershire, UK). The powder morphology and the structures of scaffolds were characterized by scanning electron microscopy (SEM; HITACHI, S4800, Tokyo, Japan) observation. The porosity (*P*) of the bioceramic scaffolds (*n *=* *3) was measured by the gravity method, according to the following equation:

$$P=\text{1}-\rho_{\text{scaffold}/}\rho_{\text{material}}$$

where *ρ*  _material_ and *ρ*  _scaffold_ are the density of low dosage of Mg-substituted wollastonite (2.91 g/cm^3^) and the apparent density of the scaffolds.

### Mechanical performance testing

The stress–strain curves of the bioceramic scaffolds (*n *=* *6) were measured using a static mechanical test machine (Instron 5566, Norwood, MA) and a 10 kN load cell, at a constant crosshead speed of 0.5 mm/min. the maximal compressive load (N) for crushing the samples while scaffold collapsing was used to compare with each other.

### Bio-dissolution evaluation in Tris buffer

According to the ratio of scaffold mass to Tris buffer volume 1.0 g/200 ml, the scaffolds (*m*_0_) were immersed in the Tris buffer with an initial pH 7.4 at 37°C. After 1, 3, 5, 7, 10 and 14 days of soaking, extracting supernatant (1.0 ml) and measuring the concentration of Ca, Mg and Si by ICP-OES, an equal volume of fresh Tris buffer (1.0 ml) was added to keep the solution volume constant. After immersion for 2, 4 and 8 weeks, the scaffolds (*n *=* *3) were rinsed with ethanol and then dried at 100°C for 12 h, and weighed (*m*_t_). The mass loss at time *t* was expressed as the equation: weight decrease = *m*_t_/*m*_0_ × 100%.

### Animal model

All animal procedures received official approval by Animal Ethics Committee at the Second Affiliated Hospital of Zhejiang University (2019-005). The male New Zealand white rabbits (4 months in age, ∼2.5–2.8 kg in mass, *n *=* *32) were randomly divided into four groups. Before surgery, all rabbits were allowed to acclimate for 7 days in stainless steel cages singly. Four sets of scaffolds that had been autoclaved were prepared for implantation *in vivo*. Prior to depilation and disinfection of the surgical area, the rabbit's ear vein was injected with 3% sodium phenobarbital at a dosage of 1 mg/kg. Subsequently, bone defects (6.5 × 5.0 × 5.0 mm) were created with the help of dental drill in the longitudinal and sagittal planes of the distal femur on both sides. And during the process of bone removal, the saline solution was continuously washed to prevent bone necrosis. The scaffolds were implanted accordingly into the defects. Finally, the wounds were sutured in layers, followed by wound disinfection. During post-operative treatment, penicillin (80 000 U) was administered once daily for 3 days and the rabbits were allowed to move freely in the cage. The rabbits were euthanized at 4, 8 and 12 weeks after implantation and the specimens were harvested for radiological and histological analysis.

### Radiologic examination

After harvesting, the femoral specimens were scanned using a special imaging system (XPERT; Kubtec Co., Stratford, CT) designed for small animals. To provide a preliminary assessment of scaffolds degradation and new bone formation, the lateral X-ray films of each group (*n *=* *4) were recorded at 45 kV and 100 μA. All of the films were recorded using a high-resolution camera (DMLA; Leica, Wetzlar, Germany). Repair effect of femoral defect was evaluated by micro-computed tomography (micro-CT; Inveon™ CT scanner, Siemens, Berlin, Germany) and all specimens were scanned vertically along the long bone axis covering the entire distal femur with a current of 80 mA and a voltage of 80 kV. That’s how the region of interest (ROI; 6.5 × 5.0 × 5.0 mm) was traced manually and virtually 3D reconstructed. For quantitative analysis, the newly formed bone (NB) volume-to-total volume (BV/TV), material residual/total volume (RV/TV) and trabecular number (Tb.N) were calculated using Inveon Acquisition Workplace (IAW; Siemens, Berlin, Germany).

### Histological observation

After the non-invasive μCT scanning, all specimens were fixed in 10% formalin for 1 week, dehydrated in graded alcohol system (70–100%) and embedded in polymethyl methacrylate (PMMA) without decalcification. Using amicrotome (SP1600; Leica, Wetzlar, Germany), the embedded specimens were cut from the middle of the defect area perpendicular to the longitudinal axis of the distal femur. Afterwards, the sections were ground and eventually polished to a thickness of 40–50 μm with a special grinding machine (Exakt-Micro-Grindin System; Leica, Wetzlar, Germany) step by step for McNeal’s trichrome staining. Finally, these stained sections were observed under a light microscope (OLYMPUS; BX53, Tokyo, Japan) at different magnifications (20×, 100×). For histomophometric analysis by a method described previously [30], pieces of high-magnification area were randomly selected from each group of specimens, the new bone (NB) area was quantitatively evaluated using ImageJ 1.46r (NIH) and BV/TV was calculated.

### Statistical analysis

All the data were expressed as mean ± standard deviation and analysed with the one-way ANOVA. In all cases, the results were considered statistically significant with a *P* value less than 0.05.

## Results

### Physicochemical characterization of core–shell bioceramic scaffolds

**Figure 1.**
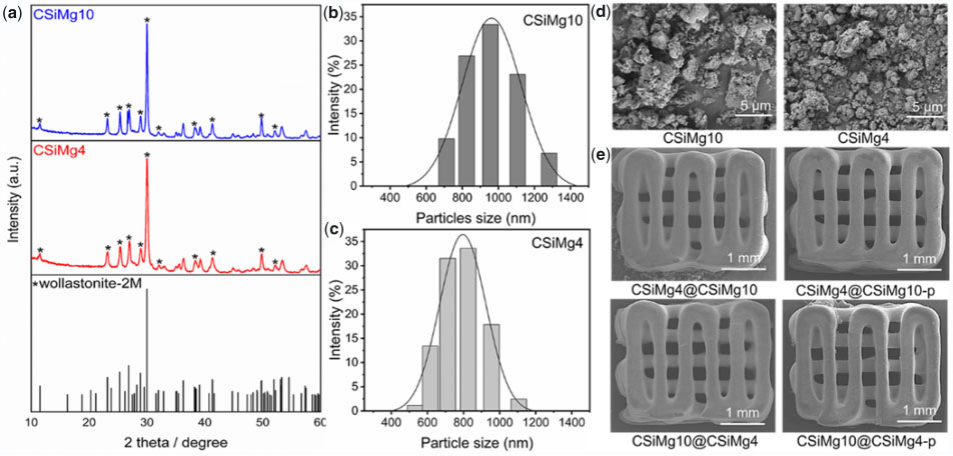
Primary characterization of the bioceramic powders and scaffolds. XRD pattern of CSiMg4 and CSiMg10 powders after calcining at 850°C, * wollastonite (**a**); particle size distribution of CSiMg4 and CSiMg10 powders (**b**, **c**); SEM images of the bioceramic powders (**d**); SEM images of the outward appearance of the bioceramic scaffolds (**e**).

According to ICP analysis, the Mg-substituting-Ca ratio in CSiMg4 and CSiMg10 were 4.13 mol% and 10.57 mol%, respectively (Table 1). The XRD patterns of bioceramic powders are shown in Fig. 1a. It was confirmed that these powders maintained the wollastonite phase (PDF# 42-0547). Meanwhile, the particle size distribution test showed that the particle size of CSiMg4 and CSiMg10 powders after milling was mainly 500–1200 and 700–1300 nm, respectively (Fig. 1b). It suggests that increasing Mg substitution ratio in wollastonite ceramic powders may readily improve sintering property but enhance the difficulty in ball milling. Meanwhile, the SEM observation also confirmed the superfine particle feature of the ground bioceramic powders (Fig. 1c and d). Moreover, the SEM images showed the typical filament morphology of the sintered samples, and the four groups of core–shell scaffolds indicated similar macropore architectures and pore interconnectivity (Fig. 1e). Table 2 lists the structural parameters of core–shell-strut bioceramic scaffolds. It was evident that the sintering linear shrinkage was very similar among the four groups of samples and the scaffolds maintained similar pore structural parameters, although the micropores in the shell layer of CSiMg10@CSiMg4-p may contribute on a slight higher porosity (52.5 ± 0.6%).

**Table 1.** ICP of 4% and 10% Mg doping CSi

	Ca (ppm)	Mg (ppm)	Si (ppm)	Mg/(Ca+Mg)
CSiMg4	1.47	0.11	1.13	4.13%
CSiMg10	38.61	1.00	28.80	10.57%

**Table 2.** Structural parameters of the core–shell bioceramic scaffolds

Group	Linear shrinkage (%)	Pore size (μm)	Apparent density, *ρ*_scaffold_ (kg/m^3^)	Porosity (%)
CSiMg4@CSiMg10	28.4 ± 1.1	∼500	1.633	46.8 ± 0.4
CSiMg4@CSiMg10-p	27.3 ± 1.9	∼490	1.530	48.3 ± 0.4
CSiMg10@CSiMg4	28.0 ± 0.9	∼500	1.598	48.0 ± 0.9
CSiMg10@CSiMg4-p	26.7 ± 0.7	∼490	1.545	52.5 ± 0.6

**Figure 2.**
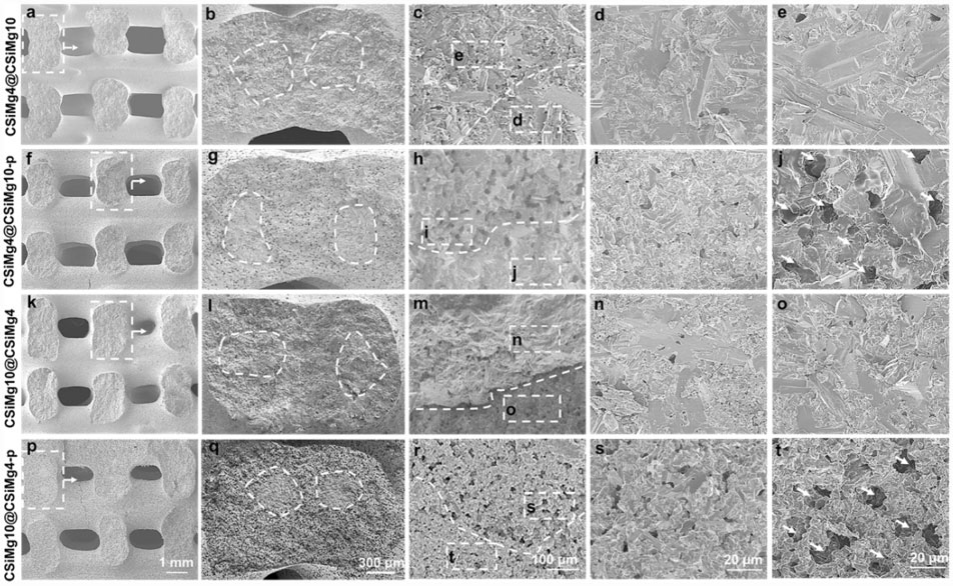
SEM Images of side-wall fracture surface of the core-shell-strut scaffolds of CSiMg4@CSiMg10 (**a–e**), CSiMg4@CSiMg10-p (**f–j**), CSiMg10@CSiMg4 (**k–o**) and CSiMg10@CSiMg4-p (**p–t**). the dotted curves represent the interface of the core/shell component and the arrows indicate the formation of microporous structures in the shell layer.

It was seen from the SEM images (Fig. 2) that the side wall in the fracture surface of scaffolds also maintained completely interconnective macropore architecture. Meanwhile, the core–shell structures in the fracture surface of the pore filaments could be observed, and especially high-density micropores in the shell layer could be found in the high-magnification SEM images in CSiMg4@CSiMg10-p and CSiMg10@CSiMg4-p (Fig. 2j and t). Although the CSiMg4 and CSiMg10 powders had similar chemical compositions, the later showed appreciable sintering and structural densification than the former based on the SEM observation for the sintered samples (CSiMg4@CSiMg10 vs. CSiMg10@CSiMg4).

### Mechanical characterization of the core–shell scaffolds

**Figure 3.**
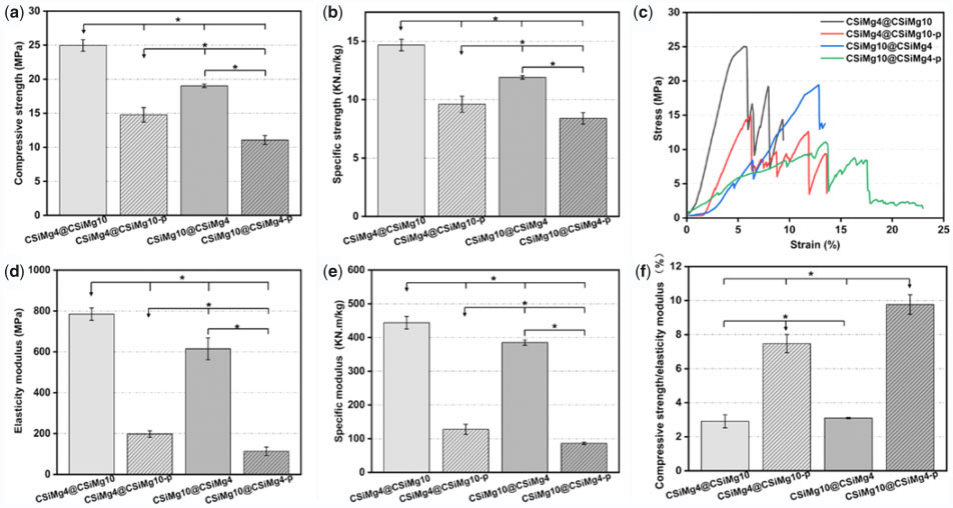
Mechanical test results of the bioceramic scaffolds including compressive strength (**a**), elasticity modulus (**b**), stress–strain curves (**c**), specific strength (**d**) and specific modulus (**e**), compressive strength/elasticity modulus (**f**). (*n *=* *6), *P < 0.05.

The mechanical properties of the bioceramic scaffolds with different composition distributions and microstructures are displayed in Fig. 3. It was evident that the CSiMg4@CSiMg10 scaffolds had higher apparent compressive strength (∼25.8 MPa) and elastic modulus (784 MPa) than the CSiMg10@CSiMg4 (∼615 MPa and 610 MPa). Meanwhile, the micropore structures in shell layer led to strength decay of the core–shell scaffolds of CSiMg4@CSiMg10-p and CSiMg10@CSiMg4-p (14.8 and 11.1 MPa). It was interesting that the apparent strength of the CSiMg4@CSiMg10 was over 2-fold higher than the CSiMg10@CSiMg4. In addition, the specific strength showed a similar difference among the scaffolds, possibly due to their similar apparent density (Fig. 3b and e). It was observed from representative stress–strain graphs that all groups of scaffolds showed a similar trend as brittle bioceramic (Fig. 3c). Especially, the loads for the CSiMg4@CSiMg10 and CSiMg10@CSiMg4 were increased almost linearly with deformation, accompanying with a typical elastic response followed by failure. As for the porous-shell scaffolds (i.e. CSiMg4@CSiMg10-p and CSiMg10@CSiMg4-p), after a linear elastic region, plateau regimes were initiated following the onset of densification and then accompanied by stress jumps in which compressive stress was reduced especially for the later. It was worth mentioning that the CSiMg10-shell scaffolds exhibited low compressive strain (∼7%) than the CSiMg4-shell scaffolds before stress decay, and the porous-shell scaffolds exhibited higher compressive strains (>14%) before full densification of porous constructs. Moreover, the ratio of strength/modulus reflected a significant difference with the strength and modulus among the scaffolds (Fig. 3f). The value for the strength-strong CSiMg4@CSiMg10 was only one-fourth of that for the CSiMg10@CSiMg4-p, implying appreciable elastic–plastic deformation ability of the later.

### Bio-dissolution behaviour of the core–shell scaffolds in vitro

**Figure 4.**
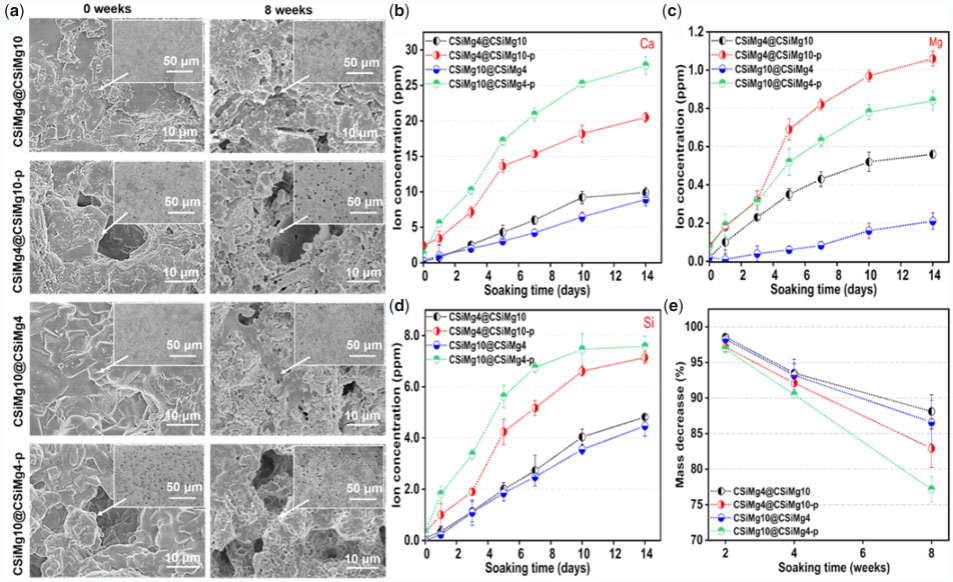
The surface microstructure of scaffolds by SEM observation before and after immersion in tris buffer (**a**) and ion release profiles of Ca (**b**), Mg (**c**) and Si (**d**) and mass loss of scaffolds (**e**) during immersion in Tris buffer for different time stages.

Figure 4a shows the surface changes of the scaffolds before and after immersion in Tris buffer for 8 weeks. It was evident that the filament surface became looser after immersion, and high-density nanopores could be observed in the shell-layer surface, regardless of the presence of PS-making micropores in the shell layer of the filaments.

As expected, the ion release behaviour of the scaffolds and the changes of ion concentrations in Tris buffers were directly correlated with the component distribution and shell-layer microstructures (Fig. 4b–d). It was shown that the scaffolds with shell-layer micropores (e.g. CSiMg4@CSiMg10-p, CSiMg10@CSiMg4-p) could contribute to a fast release of Ca, Mg and Si ions. Meanwhile, the increase of Mg content in the shell layer could also adjust the Mg ion release (Fig. 4c). It was indicated that high dissolvable porous CSiMg4 shell was optimal for accelerating Ca and Si ion release, but in comparison with the micropore-free CSiMg10@CSiMg4 scaffolds, the sparingly dissolvable CSiMg10 shell in the CSiMg4@CSiMg10 did not affect the bio-dissolution on the surface layer in the early stage (14 days; Fig. 4b and 4d). The mass decrease of the scaffolds also showed a similar trend with the ion release behaviour (Fig. 4e); that is, the micropore-rich CSiMg4@CSiMg10-p and CSiMg10@CSiMg4-p scaffolds showed significantly fast mass loss (∼17–23%) after 8 weeks, but the other two types of scaffolds free of shell-layer micropores indicated similar slower bio-dissolution and mass decrease (<13%) in the whole long stage.

### Stimulating osteogenic capability *in vivo*

**Figure 5.**
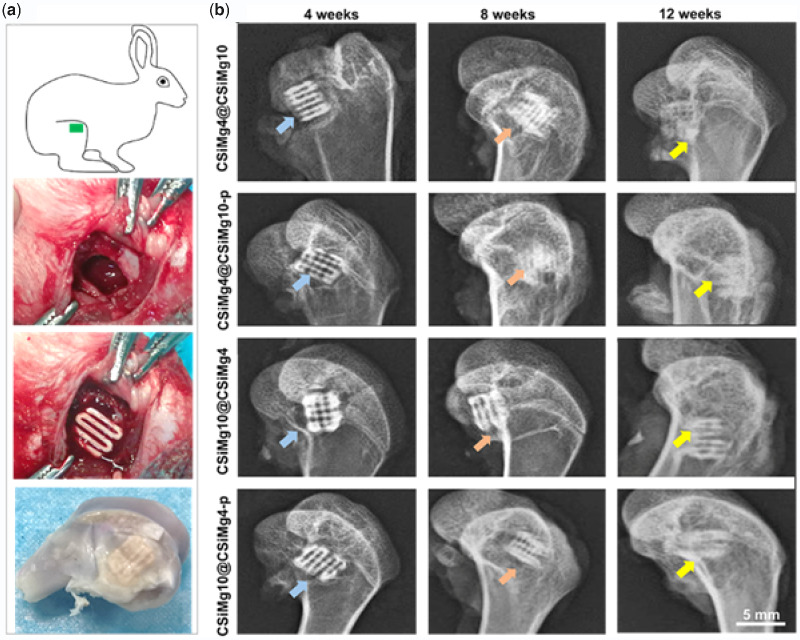
Implantation operation in femoral bone defect and the harvested femoral specimen (**a**) and radiological images of the femoral bone specimens at different time points (**b**).

As depicted in Fig. 5a, the implantation site of the scaffolds, surgical procedure and the harvested specimens. One week after surgery, the general mental state, activity and diet of the rabbits returned to normal and the operation area were clean and free of swelling and inflammatory exudation. After implantation for 4, 8 and 12 weeks, X-ray photograph analysis is shown in Fig. 5b. During the early stage of implantation (4 weeks), it could be clearly observed the interface between the scaffold and host bone tissue in each group. With the prolongation of implantation (4–12 weeks), there was increased bone integration, and it could be observed the new bone tissue ingrowth and material biodegradation. In particular, the CSiMg10@CSiMg4-p and CSiMg4@CSiMg10-p groups showed significantly higher scaffold degradation and new bone formation at 8 and 12 weeks of implantation.

**Figure 6.**
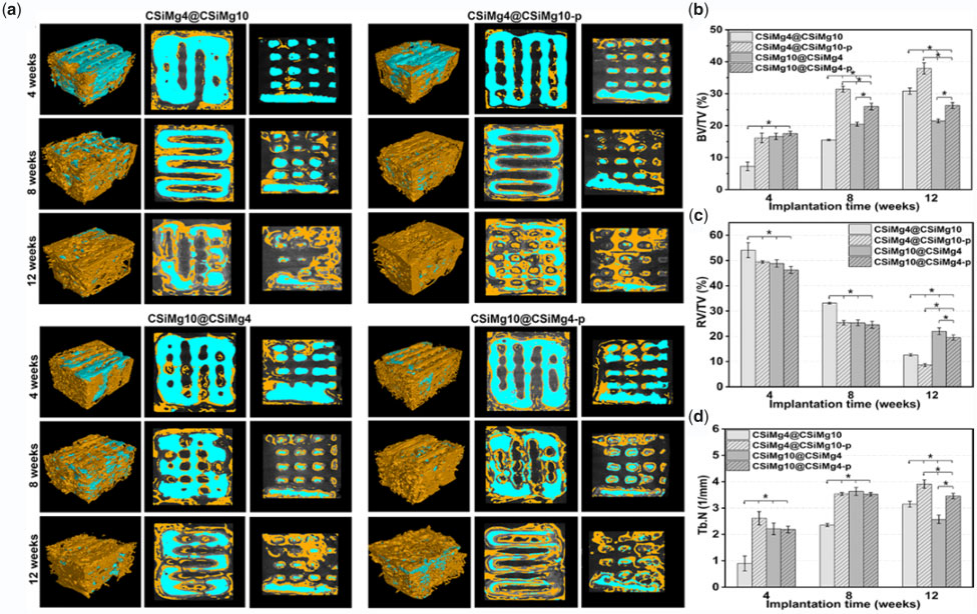
3D and 2D micro-CT images for the animal model filled with bioceramics scaffolds (**a**) and quantitative analyses of BV/TV (**b**), RV/TV (**c**) and Tb.N (**d**) based on micro-CT. Blue: bioceramics; yellow: new bone. (*n *=* *4), * *p* < 0.05.

Figure 6a shows the representative images of 2D, 3D μCT-reconstructed bone defects implanted with core–shell scaffolds at different time points. The 2D/3D images demonstrated that the bioceramic scaffolds were well implanted into the femoral defects and the scaffold biodegradation and osteogenesis were distinctly different among the different groups. The new bone initially grew along the edges and macropores of the scaffold, and then grew into the internal pore networks of the scaffold as the material degraded. There was limited osseointegration between the porous implants and the host bone in group CSiMg4@CSiMg10 at 4 weeks, and the material degradation remained stable after 8 weeks. However, the scaffold structure showed some degree of collapse but the new bone tissue could grow into the defect at 12 weeks. In contrast, the CSiMg4@CSiMg10-p group showed higher osteogenic activity and some new bone tissue could grow into the scaffolds, and especially appreciable amount of new bone tissue was formed after 12 weeks, but the material structures were maintained well. As for the two groups filled with the CSiMg10@CSiMg4 and CSiMg10@ CSiMg4-p scaffolds, the former showed slower biodegradation and the later higher new bone tissue ingrowth with time.

On the other hand, the quantitative BV/TV, RV/RV and Tb.N analysis (Fig. 6b–d) involving new bone tissue formation and material residual in the bone defects was consistent with μCT-reconstructed observation. At 4 weeks, there were no significant differences in the material degradation and new bone tissue formation except the CSiMg4@CSiMg10 group. The CSiMg4@CSiMg10 showed the lowest BV/TV and Tb.N values within 8 weeks, but the material degradation was accelerated and new bone formation was increased significantly at 12 weeks. The CSiMg4@CSiMg10-p group displayed the highest BV/TV and Tb.N value in comparison with other groups at 8 and 12 weeks. However, the BV/TV and RV/TV values for the CSiMg10@CSiMg4 and CSiMg10@CSiMg4-p groups were very limitedly changed at 8–12 weeks compared to the other two groups of scaffolds.

**Figure 7.**
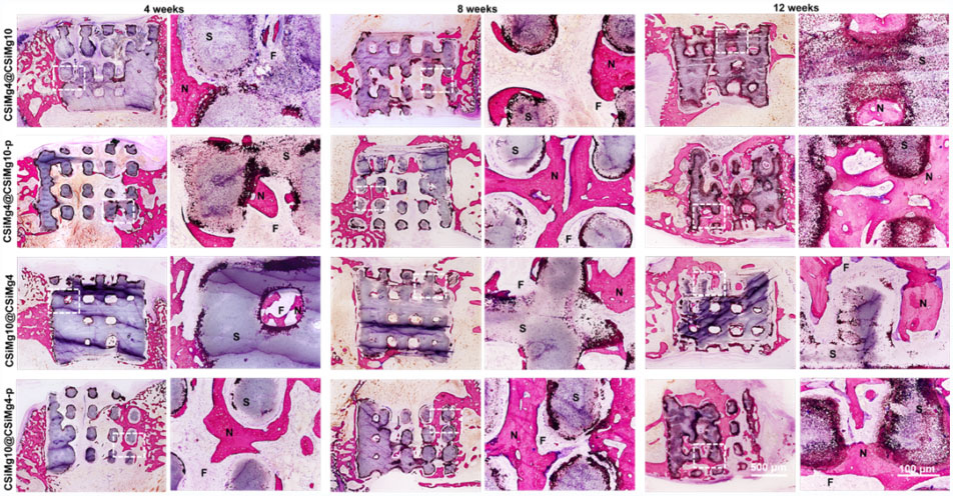
Histological analysis of McNeal-stained (20×, 100×) bone specimens at 4–12 weeks postoperatively. N: new bone, S: scaffold, F: fibre.

The histological sections of McNeal-stained specimens at 4–12 weeks are shown in Fig. 7. All core–shell bioceramic scaffolds maintained the initial pore architectures without inflammation reaction and only a small amount of new bone was formed where the scaffold contacts the host bone after 4 weeks. The new bone mainly grew adjacent to the macropore of core–shell scaffolds and some grew into the internal macropores with the partial material degradation at 8 weeks post-operatively. Compared with the other groups of scaffolds, the CSiMg4@CSiMg10-p scaffolds had a large number of mature bone tissue evenly distributing in the interconnected macropores and the scaffolds were significantly biodegradation after 12 weeks.

**Figure 8.**
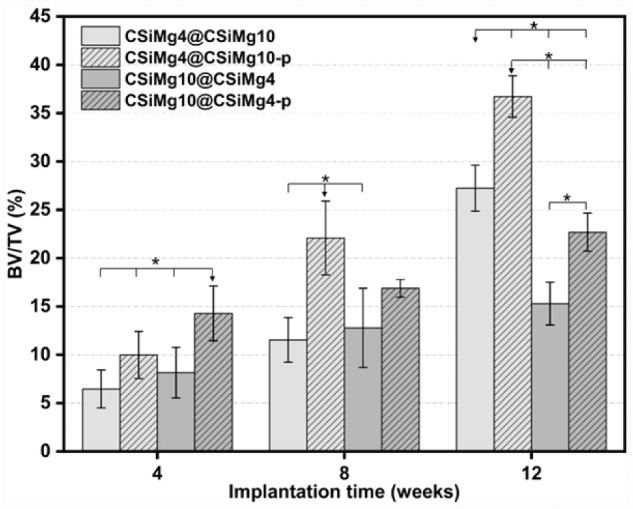
The percentage of new bone area at different stages of implantation in each group using histomophometric analysis. (*n *=* *4), * *P* < 0.05.

Histomorphometric analysis (Fig. 8) further confirmed that the new bone area in the scaffolds was increased with prolonging the implantation time. At 4 weeks, the new bone area of CSiMg10@CSiMg4-p scaffolds reached up to 14.3%, which is higher than the other three groups (≤∼10%). However, the new bone growth was significantly increased for the CSiMg4@CSiMg10-p group at 8 weeks and 12 weeks. Totally speaking, the CSiMg10@CSiMg4 and CSiMg10@CSiMg4-p groups showed slow increase of the newly formed bone formation, and, at 12 weeks, it only reached up to ∼15% and 23%, respectively. New bone formation in the CSiMg4@CSiMg10-p and CSiMg4@CSiMg10 groups was slow in the prophase and increased obviously at 12 weeks, indicating that these two groups of scaffolds are optimal for stimulating osteogenesis during the late stages.

## Discussion

It is well known that ideal porous scaffolds should provide temporary mechanical support and mass transport to stimulate cell adhesion, proliferation and differentiation, and adapt to the shape and geometry of bone defects, preventing invagination of adjacent tissues and initiating tissue healing [31, 32]. However, the comprehensive properties of the temporal scaffold are affected by the material composition and (micro)structure [33]. It has not been fully studied to achieve optimal bone regeneration via simultaneously controlling the composition distribution and structural design of different material components [20]. Fortunately, the core–shell-typed (biphasic) bioceramic microspheres via a coaxially aligned multilayer nozzle system suggested that the synchronous design of bioceramic component distribution and even microporous structures in specific component layer can effectively control their bioactive and biodegradable properties [20] and provide superior osteogenic activity in bone defect environment [21–23].

In this study, the nCSis containing different Mg contents (CSiMg4, CSiMg10) which had different biodegradation were integrated into the core or shell layer of the pore filaments, and PS microbeads could be introduced to the shell bioceramic slurry to produce high-density micropores. Foreign ion-doping nonstoichiometric wollastonite (nCSi) ceramic is widely investigated for bone scaffold design [12, 34, 35]. Our previous studies have showed that the osteogenic capacity of CSiMg10 scaffolds after 16 weeks was significantly higher than β-tricalcium phosphate and pure CSi scaffolds [36]. The mechanical strength and bioactive ions release of the core–shell nCSi scaffolds were controlled by adjusting the shell-layer micropores and CSiMg4 and CSiMg10 distribution. The animal experiment results demonstrated that the endogenous regeneration ability could be improved by adjusting the physicochemical characterizations of scaffolds to acclimatize the microenvironment of damaged bone tissues.

Recently, researchers have developed multi-scale porosity scaffolds, and filaments/wall between the macropore and micropore (<50 μm) in the filaments/wall compared with only macroporous scaffolds, the presence of microporous structures can regulate the bioactivity of materials and the microenvironment of bone defects and multi-scale porosity could promote the macropore bone healing [29, 37]. It is the reason why the macroporous scaffolds with high-density micropores are more likely to form bone *in vivo* than the micropore-free scaffolds in the early stages of osteogenesis (Fig. 6b). In fact, the CSiMg10@CSiMg4-p scaffolds indicate good osteogenesis due to rapid degradation of CSiMg4 component and higher porosity (52.5 ± 0.6%) in the early stage. It is reasonable to assume that there are three types of micro-/macro-pores in the CSiMg10@CSiMg4-p and CSiMg4@CSiMg10-p. On the one hand, the macropores with nearly 500 μm in size were maintained in the nCSi scaffolds based on the 3D scaffold model. On the other hand, the micropores in the shell layer of bioceramic struts could be retained after PS volatilization. According to previous studies, only 6–10% Mg was substituted in CSi can significantly enhance the sintering densification of the ceramic at 1150°C [15]. Thus, the CSiMg4 bioceramic is thought to be of higher undersintering at 1100°C in comparison with the CSiMg10, and it is probable that some irregular open micropores in the CSiMg4 shell layer could also contribute the porosity of the CSiMg10@CSiMg4-p scaffolds. However, the bone regeneration rate was possibly retarded due to slower biodegradation of CSiMg10 core in the later stage. In general, the increased porosity is beneficial for bone tissue ingrowth, whereas the fast decay in mechanical properties possibly lead to the collapse of porous structural integrity in the scaffold. Thus, the poor mechanical stability is also not conducive to a sustained osteogenesis. In fact, the exact porosity cannot be used as a general guide for optimal bone regeneration results because of the wide range of bone characteristics and the diversity of biomaterial structural parameters (pore morphology, pore size, pore interconnectivity) and cells should be concerned *in vivo* [38].

As for the CSiMg4@CSiMg10-p scaffolds, on the one hand, the shell-layer micropores may accelerate the bioactive ion release, and, on the other hand, the sparingly biodegradation of the CSiMg10 shell layer may maintain the stability of macropore structures in the early stage; hence, the CSiMg4@CSiMg10-p scaffolds display appreciable osteogenic capability in the whole implantation time stage. Although the increase of micropores in the shell layer of the pore filaments will weaken the mechanical strength of scaffolds, such microporous structure in filaments is conversely beneficial for improving the plastic deformation and reducing brittleness of the porous bioceramic networks, and, thus, the new bone tissue could readily form in the whole pore architectures [25].

What is important in clinical applications is that the bone implant is able to withstand pressure from surrounding tissues, such as muscles, skin and ligaments; otherwise, the results of bone grafting may be aesthetically or functionally inadequate [39]. One of the main drawbacks of bioceramics is their brittle nature, therefore, are not suitable for load bearing applications [14]. The core–shell-filament nCSi scaffolds are quasi-biphasic composite which can improve the mechanical properties of each component. The compressive pressure of the core–shell nCSi scaffolds whose shell is CSiMg10 is higher than that of the scaffolds whose shell is CSiMg4. This is mainly attributed to the shell-layer content that is nearly 3-fold higher than the core layer in the pore filament. Xie et al. have confirmed that the mechanical strength and fracture toughness of CSiMg10 ceramics are significantly higher than that of CSiMg3 ceramics [15]. Our results manifested that the compressive strength of the core–shell nCSi scaffolds (12–32 MPa) similar to that of natural cancellous bone, and meanwhile the component distribution and porous microstructure in pore filaments could modulate the mechanical properties of the scaffolds (Fig. 3a). It is evident that the mechanical properties of core–shell nCSi scaffolds were reduced by the introduction of micropores, which was consistent with other researches [28, 40]. In fact, it is valuably mentioned that the core–shell-typed nCSi distribution and selective micropore structure in shell layer is specifically favourable for enhancing the plastic deformation of the scaffolds (Fig. 3c). However, the single phase porous nCSi scaffolds only exhibited elastic response before collapse of the scaffold structure [41].

Mass loss in aqueous solution is a common index to evaluate the biodegradation potential of bioceramic scaffold *in vitro*. There was no significant difference in the bio-dissolution rate of all scaffolds in the early stage, but the difference in mass loss is increased with time (Fig. 4e). Strong mass decrease was observed on the scaffolds with shell-layer micropores, even though all the scaffolds displayed some degree of mass decay. The SEM observation also confirmed the appearance of high-density nanopores on the surface layer of dense ceramic filaments for a long-time stage. Although the process of natural tissue repair and regeneration is controlled by various glycoproteins, signalling molecules, cytokines and mechanical signals in the extracellular matrix, biomaterial scaffold with controllable properties and bioactivity could also provide biological clues to adjust cell behaviour and tissue regeneration [42–44]. Biodegradable ceramics release bioactive ions through degradation to regulate cellular behaviour and bone regeneration [12, 35]. The stimulating osteogenic potential of shell-porous CSiMg4@CSiMg10-p and CSiMg10@ CSiMg4-p was excellent in the early stage, which may be attributed to the synergistic osteo-stimulating effect of appreciable dosage of Ca, Si and Mg ions (Figs 4b–d and 6b).

Conventionally, it is feasible to design adjust composition gradient distribution and tailorable biodegradation to stimulate bone regeneration. In comparison with the other core–shell nCSi scaffolds, the CSiMg4@CSiMg10 scaffolds exhibited inferior osteogenic effect possibly due to slow bioactive ion release and osteo-stimulation in the early stage. However, once the highly dissolvable CSiMg4 core layer was exposed in the tissue environment, high dosage of Ca, Si and Mg could enhance the new bone tissue ingrowth during 8–12 weeks. In contrast, the fast biodegradation of CSiMg4 shell in CSiMg10@CSiMg4 scaffolds may contribute on the bone formation *in vivo* in the early stage, but sparingly biodegradation of the CSiMg10 core layer is unfavourable for bone regeneration at the later time stage (Fig. 6a–c). Therefore, the shell-layer micropores could not only adjust the mechanical properties but also tune the bone regeneration efficiency in the early stage. Once the pore-forming agent is introduced into the shell-layer bioceramic slurry, the tuned scaffold’s biodegradation is more suitable for the bone formation. Notably, the trabecular numbers of CSiMg10@CSiMg4 and CSiMg10@CSiMg4-p scaffolds keep on increasing at all the time point but showed a decreasing trend at 12 weeks. It was reported that during bone remodelling, bone structural units consisting primarily of bone trabeculae are likely to be absorbed prior to completion of secondary mineralization when bone turnover is high [45]. New bone must be nourished by angiogenesis and angiogenesis precedes bone regeneration, future research may focus on other biologically functional ion (e.g. Sr, Zn, Cu) selective doping in Ca-silicate and/or Ca-phosphate bioceramic components to endow with appreciable anti-infeciton potential and even promote vascularizaion and bone regeneration in pathological bone defect condition by regulating the chemical composition, component distribution and microstructure of biomaterials.

## Conclusion

In summary, this study showed that it is facile and versatile to fabricate customized scaffolds by combining 3D printing technique with coaxially aligned extruding bi-nozzles to adjust the mechanical properties and allow for a precisely tailored bioactive ion release. The mechanical and biodegradable properties and osteostimulating activity of such core–shell-typed bioceramic scaffolds were evaluated systematically, and the CSiMg4@CSiMg10-p exhibited appropriate mechanical properties and expected biodegradation behaviour while obtaining good stimulation effect of osteogenesis. Therefore, it is very promising for developing some new types of bioceramic (composite) scaffolds with customization, selective foreign ion doping, adjustable component distribution and microporous structure for bone repair and reconstruction, especially for some complex-shaped pathological bone defect environments.
